# Highly sensitive low-frequency-detectable acoustic sensor using a piezoresistive cantilever for health monitoring applications

**DOI:** 10.1038/s41598-023-33568-3

**Published:** 2023-04-20

**Authors:** Yuki Okamoto, Thanh-Vinh Nguyen, Hidetoshi Takahashi, Yusuke Takei, Hironao Okada, Masaaki Ichiki

**Affiliations:** 1 National Institute of Advanced Industrial Science and Technology (AIST), Sensing System Research Center, Tsukuba, 305-8564 Japan; 2 Department of Mechanical Engineering, Keio University, Yokohama, 223-8522 Japan

**Keywords:** Mechanical engineering, Acoustics

## Abstract

This study investigates a cantilever-based pressure sensor that can achieve a resolution of approximately 0.2 mPa, over the frequency range of 0.1–250 Hz. A piezoresistive cantilever with ultra-high acoustic compliance is used as the sensing element in the proposed pressure sensor. We achieved a cantilever with a sensitivity of approximately 40 times higher than that of the previous cantilever device by realizing an ultrathin (340 nm thick) structure with large pads and narrow hinges. Based on the measurement results, the proposed pressure sensor can measure acoustic signals with frequencies as low as 0.1 Hz. The proposed pressure sensor can be used to measure low-frequency pressure and sound, which is crucial for various applications, including photoacoustic-based gas/chemical sensing and monitoring of physiological parameters and natural disasters. We demonstrate the measurement of heart sounds with a high SNR of 58 dB. We believe the proposed microphone will be used in various applications, such as wearable health monitoring, monitoring of natural disasters, and realization of high-resolution photoacoustic-based gas sensors. We successfully measured the first (S1) and second (S2) cardiac sounds with frequencies of 7–100 Hz and 20–45 Hz, respectively.

## Introduction

Microphones, which convert sound waves to electrical signals, are indispensable in various applications, such as consumer electronics^1–5^, automobiles^6^, hearing aid devices^7,8^, wearable health monitoring^9–19^, photoacoustic-based gas sensing^20–25^, and the monitoring of natural disasters, such as volcanic eruptions^26–28^, debris flow^29^, and earthquakes^30^. Two widely used types of microphones exist, traditional electret condenser and microelectromechanical systems (MEMS)-based microphones. Compared with electret condenser microphones, MEMS-based microphones offer better performance at a smaller size^1^; thus, they are more suitable in applications where the size of the microphone is a critical design parameter, such as in smartphones, in-ear headsets, wearable devices, and gas-sensing devices.

A major factor that determines the performance of a microphone is the signal-to-noise ratio (SNR), which also indicates the smallest sound pressure that can be detected by the microphone. Significant efforts have been made to improve the SNR of MEMS-based microphones in the recent past, and conventional MEMS-based microphones have an SNR as high as 74 dB^31^, which is equivalent to a minimal detectable sound pressure of approximately 0.32 mPa. Notably, the high SNR of a conventional MEMS-based microphone is obtained only in the audible frequency range (20 Hz–20 kHz), which is the measurement target in most consumer electronics and automotive applications. However, the need for MEMS-based microphones with a high SNR over the low-frequency range (<20 Hz) has recently increased. For example, the frequency of sounds associated with natural disasters is often in the infrasound frequency range (0.01–20 Hz). In health monitoring applications, the desired frequency ranges are 10–250 Hz^13^ for heart sounds and 1–30 Hz for seismocardiography^32^. In addition, sounds related to respiratory activities can have a frequency component lower than 1 Hz^16,33^. In non-resonant photoacoustic-based gas sensors^21,34^, because the photoacoustic pressure increases as the modulation frequency of the incident light decreases^35^, improving the SNR of sound detection at low frequencies results in a high SNR of these sensors. Unfortunately, for frequencies below 20 Hz, the SNR of conventional MEMS-based microphones decreases significantly as the sound frequency decreases^36–39^.

**Figure 1:**
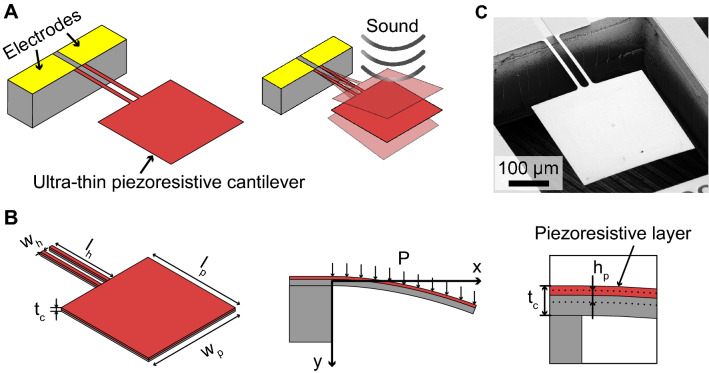
(**A**) Conceptual illustration of the proposed microphone using a piezoresistive cantilever. (**B**) Definition of the design parameters of the cantilever. (**C**) SEM image of the fabricated cantilever.

Thus, this study aims to realize a MEMS-based microphone with a high SNR over the low-frequency range of 0.1–250 Hz. The proposed MEMS-based microphone is based on a nanometer-thick piezoresistive cantilever, as shown in Fig. 1A. When sound pressure is applied to the cantilever, it bends, resulting in a resistance change. Therefore, sound pressure can be detected by measuring the resistance of the cantilever. In previous studies, piezoresistive cantilevers were used to measure the differential pressure and low-frequency sound^27,40–42^. These cantilevers can maintain a flat frequency response even at frequencies below 1 Hz^27,41^. However, the SNR of previous piezoresistive cantilevers is still poor owing to its low resolution in response to differential pressure (approximately 20 mPa). In this study, by designing a cantilever with a large pad and narrow, long hinges, high sensitivity of the cantilever in response to sound pressure can be realized, and the minimum detectable pressure as low as 200 $\upmu$ Pa can be achieved for frequencies as low as 1 Hz. This study investigates the design, fabrication, and performance evaluation of the proposed microphone. Moreover, we demonstrate the measurement of heart sounds using the prototype device.

## Design 

The design parameters of the cantilever are shown in Fig. 1B. When a differential pressure $\Delta P$ is applied to the cantilever, the bending moment caused by the applied pressure *M*(*x*) and the area moment of the cantilever *I*(*x*) are expressed as follows:

1 $$\begin{aligned} M(x)&= {\left\{ \begin{array}{ll} \frac{\Delta P w_p}{2}[2l_p(l_h-x)+l_p^2]+\Delta P w_h (l_h-x)^2 &{} (0 \le x \le l_h)\\ \\ \frac{\Delta P w_p \left( l_h + l_p - x\right) ^2}{2} &{} (l_h \le x \le l_h + l_p) \end{array}\right. } \end{aligned}$$

2 $$\begin{aligned} I(x)&= {\left\{ \begin{array}{ll} \frac{w_h t_c^3}{6} &{} (0 \le x \le l_h)\\ \\ \frac{w_p t_c^3}{12} &{} (l_h \le x \le l_h + l_p) \end{array}\right. } \end{aligned}$$

where $t_c$, $w_h$, $w_p$, $l_h$, and $l_p$ are the thickness of the cantilever, width of the cantilever hinge, width of the cantilever’s pad, length of the cantilever hinge, and length of the cantilever’s pad, respectively. The cantilever deformation profile for a minor deflection *y*(*x*) is expressed as follows:

3 $$\begin{aligned} \frac{d^2 y}{d x^2} = \frac{M(x)}{E \cdot I(x)} \end{aligned}$$

where *E* is Young’s modulus of the cantilever material. The boundary conditions when the x-axis originates from the fixed end of the cantilever, as shown in Fig. 1, are expressed as follows:

4 $$\begin{aligned}&\frac{dy(0)}{dx} = 0 \end{aligned}$$

5 $$\begin{aligned}&y(0) = 0 \end{aligned}$$

    The deflection *y*(*x*) and the spring constant *k* can be calculated from these equations. From Eqs. (1), (2), (3), and (5), the deflection of the cantilever *y*(*x*) when the constant pressure *ΔP* is applied to the surface is described as follows:

6 $$\begin{aligned} \frac{E t_c^3 w_h {\left( x \right) }}{6} \frac{d^2 y}{d x^2}&= \Delta P w_h \left( l_h - x\right) ^{2} + \frac{\Delta P w_p \left( - l_p^2 + 2 l_p \left( l_h + l_p - x\right) \right) }{2}&(0 \le x \le l_h) \end{aligned}$$

7 $$\begin{aligned} \frac{E t_c^3 w_p {\left( x \right) }}{12} \frac{d^2 y}{d x^2}&= \frac{\Delta P w_p \left( l_h + l_p - x\right) ^2}{2}&(l_h \le x \le l_p + l_h) \end{aligned}$$

    Therefore, from Eqs. (6) and (7), the deflection *y*(*x*) can be mathematically derived as follows:

8 $$\begin{aligned} y(x)&= {\left\{ \begin{array}{ll} \frac{\Delta P x^{2} \cdot \left( 6 l_{h}^{2} w_{h} - 4 l_{h} w_{h} x - 2 l_{p} w_{p} x + 3 l_{p} w_{p} \cdot \left( 2 l_{h} + l_{p}\right) + w_{h} x^{2}\right) }{2 E t_c^{3} w_{h}} &{} (0 \le x \le l_h)\\ \frac{\Delta P (2 l_{h}^{2} l_{p} w_{h} \cdot (2 l_{h} + 3 l_{p}) - l_{h}^{2} l_{p} w_{p} \cdot (2 l_{h} + 3 l_{p}) - 12 l_{h} l_{p} w_{h} x (l_{h} + l_{p}) + 6 l_{h} l_{p} w_{p} x (l_{h} + l_{p}) + w_{h} x^{4} - 4 w_{h} x^{3} (l_{h} + l_{p}) + 6 w_{h} x^{2} (l_{h}+l_{p})^2)}{2 E t_c^{3} w_{h}} &{} (l_h \le x \le l_h + l_p) \end{array}\right. } \end{aligned}$$

    The maximum deflection of the cantilever at $x = l_h+l_p$ is

9 $$\begin{aligned} d = y(x=l_h+l_p) = \frac{\Delta P \left( l_{h} l_{p} w_{p} \cdot \left( 4 l_{h}^{2} + 9 l_{h} l_{p} + 6 l_{p}^{2}\right) + w_{h} \cdot \left( 3 l_{h}^{4} + 4 l_{h}^{3} l_{p} + 3 l_{p}^{4}\right) \right) }{2 E t_c^{3} w_{h}} \end{aligned}$$

    Therefore, using Eq. (9), the spring constant when constant pressure is uniformly applied to the cantilever is expressed as follows:

10 $$\begin{aligned} k = \frac{\Delta P(2 w_h l_h + w_p l_p)}{d} = \frac{2 E t_c^{3} w_{h} \cdot \left( 2 l_{h} w_{h} + l_{p} w_{p}\right) }{l_{h} l_{p} w_{p} \cdot \left( 4 l_{h}^{2} + 9 l_{h} l_{p} + 6 l_{p}^{2}\right) + w_{h} \cdot \left( 3 l_{h}^{4} + 4 l_{h}^{3} l_{p} + 3 l_{p}^{4}\right) } \end{aligned}$$

    Conversely, from^43^, the spring constant *k* when a concentrated load is applied at the tip of the cantilever is expressed as:

11 $$\begin{aligned} k = \frac{Ew_p w_h t_c^3}{4w_h l_p^3+2t_c(3 l_h^2 l_p +3 l_h l_p^2 + l_h^3)} \end{aligned}$$

    As a dynamic property, the first resonance frequency of the cantilever is expressed as:

12 $$\begin{aligned} f_r = \frac{1}{2\pi }\sqrt{\frac{k}{m_{eff}}} \end{aligned}$$

where *k* and $m_{eff}$ are the spring constant and effective mass of the cantilever, respectively. In this structure, $m_{eff}$ is expressed as 0.24*m*, where *m* is the cantilever mass. Considering the damping ratio in air, the resonant frequency in air, $f_d$ is expressed as:

13 $$\begin{aligned} f_d = \sqrt{1-\xi ^2}f_r \end{aligned}$$

    The design parameters of the cantilever used in this study are listed in Table 1. As observed in equation (9), the wider square pad ($w_p$ and $l_p$) should be designed for larger displacement per the same applied pressure ($\Delta P$). However, these increases also result in the resonant frequency reduction, as observed in Eqs. (10), (12), and (13). In our target of heart-sound monitoring, the maximum frequency is less than 300 Hz^44^. Therefore, We designed $w_p$ and $l_p$ to be sensitive to signals up to 300 Hz and three times larger than in previous studies reported by Takahashi et al*.*^40^ to obtain larger signals.

**Figure 2:**
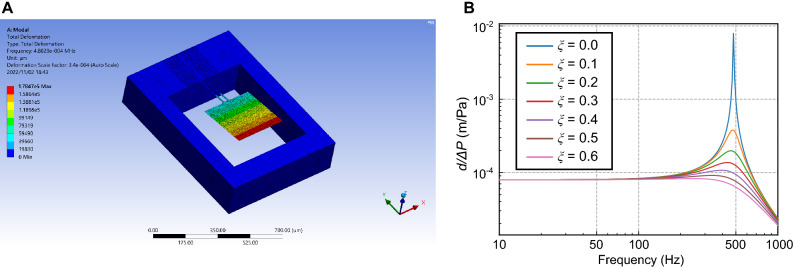
(**A**) FEM simulation of the proposed cantilever. (**B**) FEM simulation results sweeping the damping factor.

As an electrical property of the piezoresistive layer, the fractional resistance change $\Delta R/R$ of the cantilever can be calculated as^40^

14 $$\begin{aligned} \frac{\Delta R}{R} = \frac{\alpha h_p}{l_h}\int _{0}^{l_h}\frac{M(x)}{I(x)}dx \end{aligned}$$

where $\alpha$ is a constant indicating the relationship between the fractional resistance change and stress, and $h_p$ is the distance between the piezoresistive layer and the neutral axis (x-axis, as shown in Fig. 1B). Inserting Eqs. (1) and (2) into Eq. (14), we obtain

15 $$\begin{aligned} \frac{\Delta R}{R} = \Delta P h_p \frac{2l_h^2w_h + 3l_p w_p (l_h + l_p)}{t_c^3 w_h} \end{aligned}$$

As observed in Eq. (15), for the same cantilever thickness $t_c$ and piezoresistive layer, the sensitivity of the cantilever $(\Delta R/R)/\Delta P$ can be improved by increasing the length $l_h$ of the cantilever hinges, the width $w_p$ and length $l_h$ of the cantilever pad, and decreasing the width $w_h$ of the cantilever hinges. In other words, high sensitivity can be achieved by designing a cantilever with long narrow hinges and a wide pad.

**Table 1:** Design parameters of the cantilever.

$w_h$	$l_h$	$w_p$	$l_p$	$t_c$
10 ${\upmu }$m	20 ${\upmu }$m	300 ${\upmu }$m	300 ${\upmu }$ m	0.34 ${\upmu }$m

Using Eq. (15) and assuming that $\alpha$ and $h_p$ are the same for both designs, the sensitivity of the designed cantilever is expected to be approximately 55 times higher than that of the previous cantilever design^40^. The finite element method (FEM) simulation results for the cantilever using ANSYS are shown in Fig. 2A. Under vacuum conditions, the resonant frequency is 498 Hz. $\xi$ was swept from 0.1 to 0.6 to investigate the damping effect. As the value of $\xi$ increases, the resonant peak flattens, as shown in Fig. 2B.

## Results

### Measurement of resonant frequency

**Figure 3:**
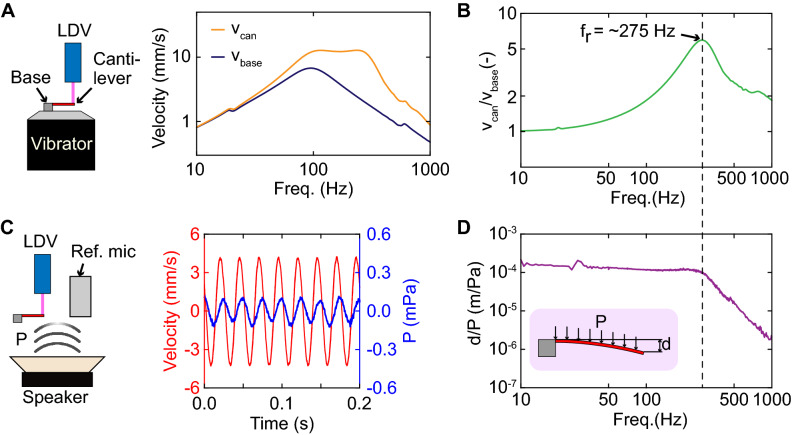
(**A**) Setup to measure the resonant frequency of the cantilever. (**B**) The frequency response characteristic of the cantilever in response to the vibration applied to the chip. $V_{\textrm{can}}$ and $V_{\textrm{base}}$ represent the vibration amplitudes of the cantilever and the chip base, respectively. (**C**) Raw data of the cantilever’s vibration and reference microphone output. (**D**) Frequency spectrum data of the cantilever’s vibration amplitude when the sound waves are applied.

**Figure 4:**
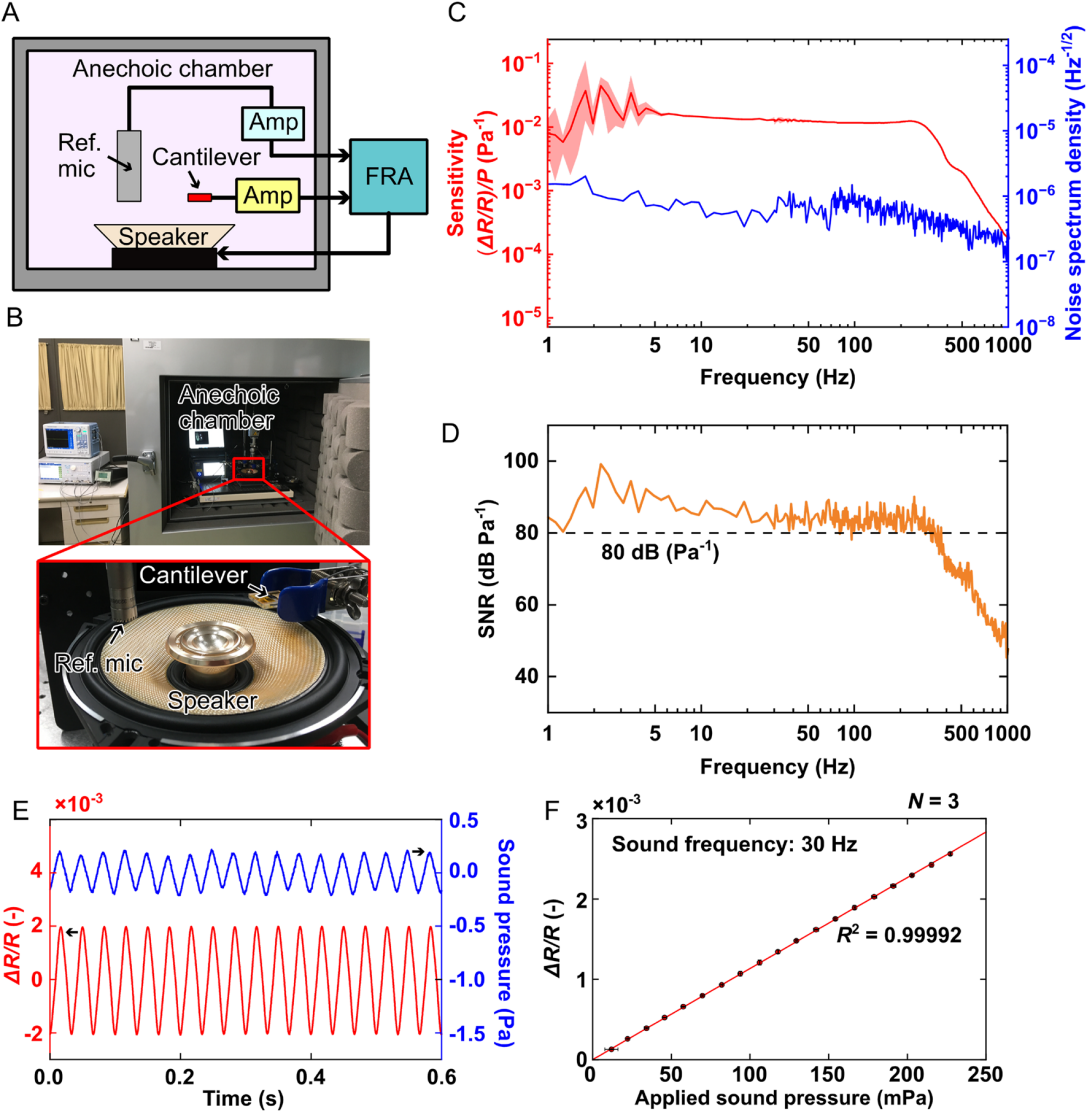
(**A**) Conceptual design of the experimental setup to evaluate the performance of the cantilever (**B**) Photographs of the experimental setup. (**C**) Measurement results of the sensitivity and noise spectrum of the cantilever output. (**D**) The resolution and SNR derived from the results in (**C**). (**E**) Raw data of the cantilever and reference microphone output. (**F**) Relationship between the resistance change of the cantilever and the applied sound pressure.

**Figure 5:**
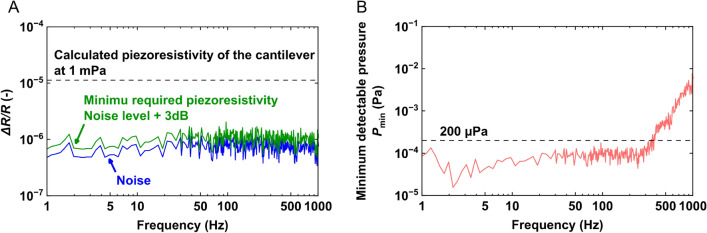
(**A**) The piezoresistivity of the noise and 3 dB higher than the noise level. The dashed line represents the calculated piezoresistivity at 1 mPa pressure (**B**) Minimum detectable pressure ($P_{\textrm{mim}}$) calculated from the sensitivity.

The first resonant frequency of the cantilever was evaluated using the setup shown in Fig. 3A. The fabricated sensor chip was attached to a vibrator (type 4810, Brüel & Kjær, Nærum, Denmark), which applied vibration in the frequency range of 10 Hz to 600 Hz to the sensor chip. A laser Doppler vibrometer (VFX-I-130, Polytec, Baden-Württemberg, Germany) was used to measure the vibrations of the cantilever surface and the base where the handle and device layer of the silicon-on-insulator wafer were not etched. As shown in Fig. 3A, the base vibrator had a resonant frequency of 100 Hz. Therefore, the cantilever’s signal also had peaks at 100 Hz. To eliminate the effect of the base vibrator’s resonance, we measured the ratio of the cantilever and the base vibrator outputs. The measurement results are shown in Fig. 3B, which describes the ratio of the cantilever’s vibration amplitude to that of the chip base. From the result, the first resonant frequency of the cantilever was determined to be approximately 275 Hz. Therefore, the cantilever is suitable for measuring sound waves with frequencies as high as 275 Hz. Then, we evaluated the cantilever’s vibration when the sound waves were applied. For the measurement, the fabricated cantilever and the reference microphone were placed at the same distance of 1 cm from a loudspeaker (KFC-XS174S, Kenwood Corp., Tokyo, Japan). The vibration of the cantilever was measured using the same vibrator. Figure 3C shows the raw data of the cantilever’s velocity and the reference microphone’s output. Figure 3D shows the frequency responses of the cantilever’s vibration amplitude when the sound waves were applied using the loudspeaker. The measurement results corresponded to the simulation results under damped conditions, as shown in Fig. 2B. We supposed the sensor was utilized in the atmosphere, and the air-damping factor was constant. fDue to the damping, we obtained flat frequency responses over a frequency range of 1–250 Hz without the sharp resonant peak.

### Performance evaluation

**Figure 6:**
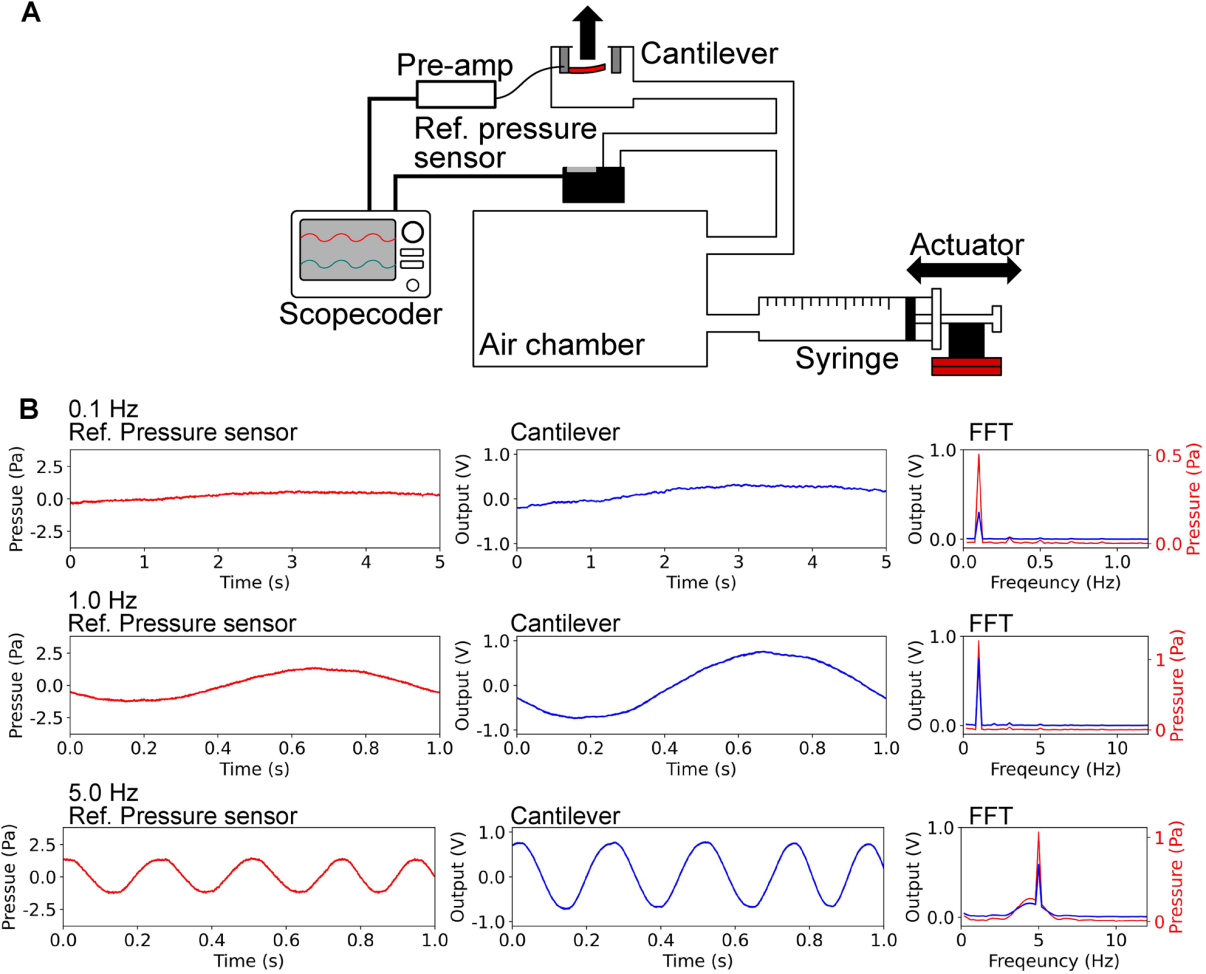
(**A**) Conceptual schematic of the experimental setup to evaluate the performance of the cantilever at low frequencies. (**B**) Timescale and frequency spectrum data of the measurement results of the reference pressure sensor and the proposed acoustic sensor.

We evaluated the sensitivity and SNR of the fabricated cantilever in response to sound waves using the experimental setup shown in Fig. 4A. Photographs of the experimental setup are shown in Fig. 4B. For the measurement, the fabricated cantilever and a reference microphone (Type 4955, Brüel & Kjær, Nærum, Denmark) were placed at the same distance of 1 cm from a loudspeaker (KFC-XS174S, Kenwood Corp., Tokyo, Japan). The evaluation setup was placed inside an anechoic chamber to reduce ambient acoustic noise. The loudspeaker was driven by applying an AC voltage from a frequency response analyzer (FRA51602, NF, Yokohama, Japan). The resistance change of the cantilever was measured using a lab-made amplifier composed of a Wheatstone bridge circuit. The resistance change of the cantilever was calculated from the output of the voltage change of the amplifier output, as follows:

16 $$\begin{aligned} \frac{\Delta R}{R} = \frac{4}{G}\frac{\Delta V}{V_0} \end{aligned}$$

where $\Delta V$ is the voltage change in the amplifier, $G = 5000$ is the amplifier gain, and $V_0 = 1$ V is the voltage applied to the Wheatstone bridge circuit. The outputs of the amplifier and reference microphone were simultaneously measured using a frequency response analyzer. The sound pressure applied to the cantilever can be calculated from the output of the microphone, and the sensitivity of the cantilever, defined as the ratio between the resistance change and applied sound pressure, can be calculated as $(\Delta R/R)/P$.

The measured sensitivity of the fabricated cantilever and its noise spectrum density (NSD) over a frequency range of 1–1000 Hz is shown in Fig. 4C. We performed the measurement five times. The red line represents the average of five measurements. The NSD was calculated as $(\Delta R/R)$ divided by the square root of the bandwidth when the speaker was switched off. In this experiment, we performed the experiment without using the laser irradiation to the cantilever inside an anechoic chamber. Besides, the power consumption in the piezoresistance in the cantilever is small enough to be ignored, and the room temperature was constant. Therefore, we consider that the dominant noise factor is caused by the amplifier. The NSD caused by the amplifier was 50 $\upmu$ V$\cdot$Hz$^{-1/2}$ at 10 Hz and is inversely proportional to the frequency of the input signal. This equivalent value converted to piezoresistive change ($\Delta R/R$) is $4.0\times 10^{-5}$ Hz$^{-1/2}$, which meets the measured NSD, as shown in Fig. 4C. Based on the sensitivity measurement result, the cantilever exhibits a flat frequency response in the frequency range of 1 to over 250 Hz. When the frequency increased in the range of over 250 Hz, the sensitivity of the cantilever started decreasing. The measured sensitivity was unstable at low frequencies (< 5 Hz). This fluctuation in the sensitivity of the cantilever in the low-frequency region resulted from the low output signal of the reference microphone and the limitation of the loudspeaker in this frequency range. Over the frequency range of 5 to 250 Hz, the average sensitivity of the cantilever was $12.4\times 10^{-3}{\textrm{Pa}}^{-1}$, approximately 40 times higher than the previous cantilever with a similar thickness^40^. This improvement in sensitivity is also of the same order as the result of the theoretical analysis described in the previous section. In contrast, the NSD of the cantilever resistance change calculated from the amplifier circuit output was generally less than $2.0\times 10^{-6}$ Hz$^{-1/2}$ over the measured frequency ranges. From the measured sensitivity and NSD, SNR is calculated as:

17 $$\begin{aligned} \textrm{SNR}&=\frac{\textrm{Sensitivity}\cdot \sqrt{f_{\textrm{BW}}}}{\textrm{NSD}\cdot \sqrt{f_{\textrm{BW}}}} \nonumber \\&\quad = \frac{\Delta R/R/P}{(\Delta R/R)_{\textrm{noise}}} \end{aligned}$$

where $f_{\textrm{BW}}$ is the measurement bandwidth. Figure 4D shows the derived SNR of the cantilever. The SNR is approximately 80 dB Pa$^{-1}$ over a frequency range of 1–250 Hz.

Moreover, we evaluated the linearity of the cantilever output by changing the applied sound pressure and measuring the change in cantilever resistance. During the measurement, the frequency of the applied sound was fixed at 30 Hz, and the voltage applied to the speaker was varied in the range of 0.5–10 V. An example of the measured data is shown in Fig. 4E. The sound pressure was calculated directly from the measured signal from the reference microphone. The relationship between the change in resistance of the cantilever and the applied sound pressure is shown in Fig. 4F. The applied pressure ranged from 0 to 250 mPa. The resistance change of the cantilever increased linearly with an increasing sound pressure with a coefficient of $11.3\times 10^{-3}\,{\textrm{Pa}}^{-1}$, which is in good agreement with the sensitivity shown in Fig. 4C.

To estimate the minimum detectable pressure, the lower limit of the detectable piezoresistive change is defined as 3 dB higher than the noise value. In Fig. 5A, the 3 dB larger piezoresistive change ($(\Delta R/R)_{\mathrm{Noise+3dB}}$) is represented by the green line, and the blue line represents the noise level. The dashed line represents the piezoresistivity calculated from result shown in Fig. 4F, when 1-mPa pressure is applied. As shown in Fig. 5A, the resistive change of the proposed sensor at 1 mPa is much higher to the minimum required value. Figure 5B shows the mimum detectable pressure ($P_{\textrm{mim}}$) calculated as:

18 $$\begin{aligned} P_{min}&= \frac{(\Delta R/R)_{\mathrm{Noise+3dB}}}{\textrm{Resolution}} \nonumber \\&\quad = \frac{(\Delta R/R)_{\mathrm{Noise+3dB}}}{(\Delta R/R/P)_{\textrm{Sens}}} \end{aligned}$$

where $(\Delta R/R/P)_{\textrm{Sens}}$ is the sensitivity of the proposed cantilever, as shown in Fig. 4C. Based on the results, $P_{\textrm{min}}$ of the proposed cantilever was approximately 200 ${{\upmu {\rm Pa}}}$ over a frequency range of 1–250 Hz, which is the highest among MEMS-based microphones.

From the results shown in Fig. 4, the sensitivity of the cantilever’s output was considerably better than that of the reference microphone’s output, particularly at low frequencies. When the frequency increased over 250 Hz, the sensitivity of the cantilever started decreasing. The measured sensitivity was unstable at low frequencies ($< 5$ Hz). For frequencies below 5 Hz, the signals of the reference microphone could not be distinguished from its noise, whereas the signal of the cantilever could be observed in the raw data and frequency spectra. Because the sensitivity of the cantilever shown in Fig. 4C was derived by dividing the output of the cantilever by that of the reference microphone, the low SNR of the reference microphone in the frequency range of 1–5 Hz caused the fluctuation in the calculated sensitivity of the cantilever over this frequency range.

**Figure 7:**
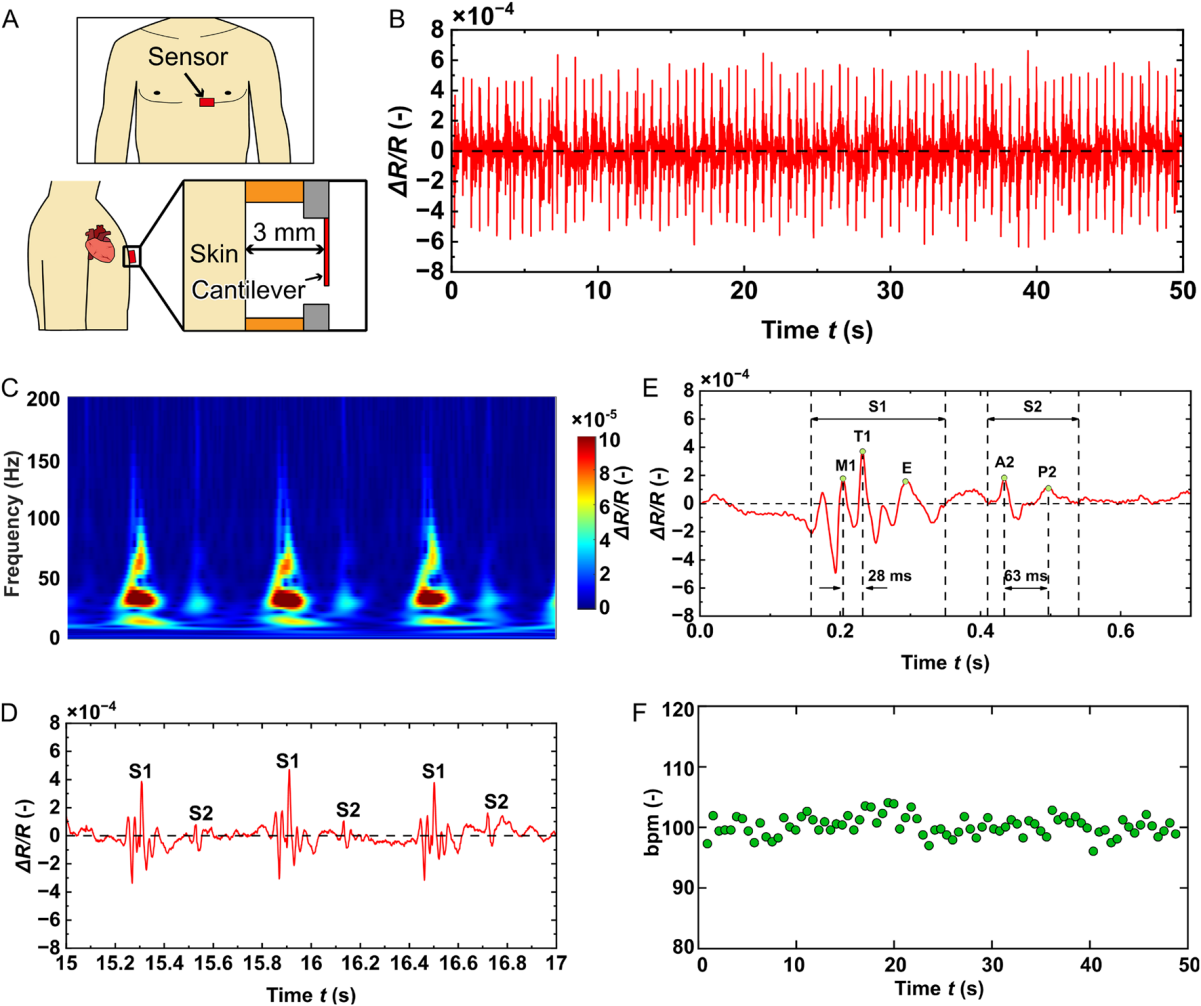
(**A**) Conceptual schematic of the proposed sensor for measuring heart sound. (**B**) Raw data of the pulse sound measurement. (**C**) Wavelet scalogram of the recorded heart sound in the duration of 15–17 s. (**D**) A zoomed-in view of the recorded heart sound in the duration of 15–17 s. (**E**) A zoomed-in view of a single cycle of the heart sound. (**F**) Heart rate of the subject for each individual heartbeat.

We performed another measurement using the differential air pressure to investigate the response of the cantilever at a frequency lower than 5Hz, as shown in Fig. 6A. The cantilever device was connected to an air chamber where the pressure was controlled using a syringe. In this measurement, the gain of amplifier *G* was set to 1000, and the voltage change of amplifier $V_0$ was set to 1 V. The differential pressure was controlled by changing the actuator position connected to the syringe. In the previous study^41^, the measurement system investigated the device characteristics at a low frequency of less than 10 Hz. We connected a reference pressure sensor to validate the application of differential pressure. The measurement results of the proposed acoustic sensor and the referential pressure sensor, as well as their frequency spectra, obtained using fast Fourier transform are shown in Fig. 6B. Based on the results, the proposed cantilever sensor is sensitive even in the low-frequency range, and the results are consistent with the results of the sound wave experiment, as shown in Fig. 4.

### Heart sound measurement

We measured heart sounds using the fabricated cantilever to demonstrate the application of the proposed microphone in health monitoring. The fabricated cantilever chip was glued to a 3D-printed jig attached to the chest of a subject (male, 37 years old), as shown in Fig. 7A. The thickness of the jig allowed the cantilever to be located approximately 3 mm above the subject’s chest. The raw data of a measurement performed for 50 s is shown in Fig. 7B. The cantilever can measure the heart sound of the subject, and the peak-to-peak amplitude of the heart sound signal was approximately $8\times 10^{-4}$ (−). As shown in Fig. 7C, the noise level of the amplifier is equivalent to a fractional resistance change of $10^{-6}$ (−); thus, the recorded heart sound signal has an SNR of 58 dB, which is 17 dB better than the measurement result obtained using a highly sensitive mechanoacoustic sensor based on the nanofiber previously reported^13^.

A zoomed-in view of the recorded heart sound in the duration of 15 to 17 s and its wavelet scalogram are shown in Fig. 7C,D shows the raw signal in the same duration. As shown in Fig. 7C,D, the signal has two dominant events. These events are well-known heart sounds, called as the first (S1) and second (S2) cardiac sounds^44,45^. S1 is generated by the closure of the mitral and tricuspid valves, and the closure of the aortic and pulmonary valves generates S2. Figure 7E shows the zoomed-in view of a single cycle of the heart sound. As shown in Fig. 7e, the major components in S1 and S2 were observed. In S1, three peaks were observed. The first two sharp peaks (M1 and T1) were caused by the closure of the mitral and tricuspid valves, respectively^44^. In this measurement, T1 was heard 28 msec later than M1. The delay is known to be smaller than 35 msec, and the value is normal. The final peak (E) was the ejection sound associated with the opening of the aortic valve. In S2, two peaks were observed. The first sound (A2) was generated by the closure of the aortic valve, and the pulmonary valve generated the second sound (P2). The delay between the A2 and P2 was 63 msec.

Moreover, from the wavelet scalogram, the dominant frequency ranges of S1 and S2 were 7 to 100 and 20 to 45 Hz, respectively. In addition, the high SNR of the recorded signal facilitated the calculation of the timing of the peak of S1 for each heartbeat. From this calculation, the heart rate of the subject can be obtained for each heartbeat, as shown in Fig. 7F. Based on the aforementioned measurement results, the proposed cantilever is an excellent candidate for wearable heart sound monitoring devices. The proposed cantilever can measure heart sounds when placed several millimeters above the skin. In other words, the cantilever must not be attached to the skin to measure the heart sound, as required by many mechanoacoustic sensors. This is advantageous for wearable health monitoring devices because the contact area between the device and the skin is significantly reduced, causing discomfort to the user.

## Methods

### Fabrication

**Figure 8:**
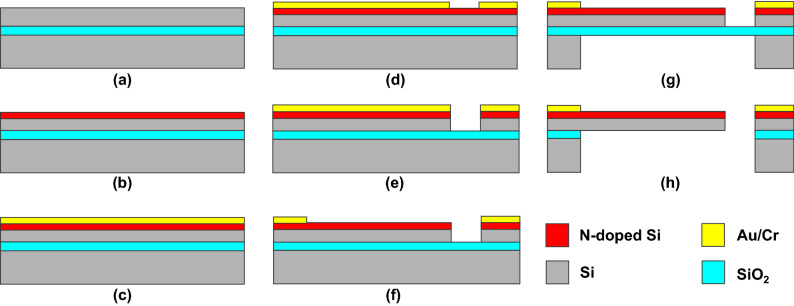
Fabrication process of the proposed cantilever-type acoustic sensor.

The fabrication process of the sensor is shown in Fig. 8. A silicon-on-insulator wafer (thickness: 0.34/0.4/250 ${\upmu }$m) was used to fabricate the sensor. First, the piezoresistive layer was formed by doping the device layer with arsenic ions via ion implantation followed by annealing (700$^\circ$C for 10 min, and by 900$^\circ$C for 30 min). Next, the oxide layer formed on the Si layer during annealing was removed by dipping the wafer in a hydrogen fluoride (HF) solution. Chromium and gold layers with thicknesses of 5 and 50 nm were deposited on the piezoresistive layer, respectively. The metal and Si layers were then etched by wet etching and deep reactive ion etching, respectively, to form the cantilever. Next, the metal layers on top of the cantilever were removed by wet etching. Finally, the cantilever was released by etching the handle Si layer and removing the box layer using HF vapor. Moreover, additional cleaning with O$_2$ plasma was applied to remove the residual photoresist and oil attached to the cantilever after removing the box layer. The SEM image of the fabricated cantilever is shown in Fig. 1C. The initial resistance of the cantilever was approximately 5.4 k$\Omega$.

### Heart sound measurement

The jig attached to the chest of a subject was fabricated using an inkjet 3D printer (AGILISTA, Keyence, Japan) with a minimum pattern width of 15 $\upmu$m. The authors declare that the jig does not have skin-irritating properties. The experimental protocols in this paper were approved by the IRB of Ethics in Ergonomic Experiments (2019-988-B) in the National Institute of Advanced Industrial Science and Technology (AIST), including any relevant details. Moreover, the authors declare that all experiments were carried out following the relevant guidance and regulations and confirm that informed consent was obtained from all participants.

## Conclusions

This study investigated the design of a MEMS-based microphone that can achieve a resolution of approximately 0.2 mPa over the frequency range of 0.1 to 250 Hz, which is the highest value reported to date for MEMS-based acoustic sensors. The high performance of the proposed microphone was enabled by the piezoresistive cantilever, which was 340 nm thick and possessed a 300 $\mu$m-wide pad and two 10 ${\upmu }$m-wide, 200 ${\upmu }$m-long hinges. This extremely flexible structure allowed the cantilever to obtain a sensitivity of over 10$^{-2}$  Pa$^{-1}$, which was 40 times higher than that of the previous cantilever design with a similar thickness. The superior SNR of the cantilever over the low-frequency sound compared with the reference commercial microphone was demonstrated through measurement results. Using the fabricated cantilever, we demonstrated the measurement of heart sounds with an SNR as high as 58 dB. We believe that the proposed microphone will be useful in various applications, such as wearable health monitoring, monitoring of natural disasters, and realization of high-resolution photoacoustic-based gas sensors.

## Data Availability

The datasets generated during and/or analysed during the current study are available from the corresponding author on reasonable request.
